# Sex-Specific Inflammatory Profiles Affect Neuropsychiatric Issues in COVID-19 Survivors

**DOI:** 10.3390/biom15040600

**Published:** 2025-04-18

**Authors:** Mariagrazia Palladini, Mario Gennaro Mazza, Beatrice Bravi, Margherita Bessi, Maria Cristina Lorenzi, Sara Spadini, Rebecca De Lorenzo, Patrizia Rovere-Querini, Roberto Furlan, Francesco Benedetti

**Affiliations:** 1 Vita-Salute San Raffaele University, 20132 Milano, Italy; delorenzo.rebecca@hsr.it (R.D.L.); rovere.patrizia@hsr.it (P.R.-Q.); furlan.roberto@hsr.it (R.F.); benedetti.francesco@hsr.it (F.B.); 2 Psychiatry and Clinical Psychobiology Unit, Division of Neuroscience, IRCCS Ospedale San Raffaele, 20132 Milano, Italy; mazza.mario@hsr.it (M.G.M.); bravi.beatrice@hsr.it (B.B.); bessi.margherita@hsr.it (M.B.); lorenzi.cristina@hsr.it (M.C.L.); spadini.sara@hsr.it (S.S.); 3 Unit of Innate Immunity and Tissue Remodelling, Department of Internal Medicine, Division of Immunology, Transplantation and Infectious Diseases, IRCCS Ospedale San Raffaele, 20132 Milano, Italy; 4 Clinical Neuroimmunology, Division of Neuroscience, IRCCS Scientific Institute Ospedale San Raffaele, 20132 Milano, Italy

**Keywords:** COVID-19, inflammation, depression, cognition

## Abstract

Post-COVID syndrome has unveiled intricate connections between inflammation, depressive psychopathology, and cognitive impairment. This study investigates these relationships in 101 COVID-19 survivors, focusing on sex-specific variations. Utilizing path modelling techniques, we analyzed the interplay of a one-month 48-biomarker inflammatory panel, with three-months of depressive symptoms and cognitive performance. The findings indicate that cognitive impairment is influenced by both inflammation and depression in the overall cohort. However, prominent sex-specific differences emerged. In females, a lingering imbalance between pro- and anti-inflammatory responses—likely reflecting the long-lasting immune alterations triggered by COVID-19—significantly affects cognitive functioning and shows a marginal, though not statistically significant, association with depressive symptoms. This suggests that a mixed inflammatory profile may contribute to these outcomes. Conversely, in males, inflammation was inversely associated with depression severity, with protective effects from regulatory mediators (IL-2, IL-4, IL-6, IL-15, LIF, TNF-α, β-NGF) against depression. In males, cognitive impairment appeared to be driven mainly by depressive symptoms, with minimal influence from inflammatory markers. These results highlight distinct sex-specific pathways in immune and inflammatory responses post-COVID-19, potentially shaped by endocrine mechanisms. The findings suggest that persistent inflammation may foster long-term neuropsychiatric sequelae, possibly through its effects on the brain, and underscore the need for sex-tailored therapeutic strategies to address the lasting impact of COVID-19.

## 1. Introduction

The relationship between inflammation, depression, and cognitive impairment has garnered increasing attention in recent years, particularly due to the complex and interrelated nature of these factors [1]. Inflammation represents a common pathway influencing both mood regulation and cognitive functioning, with cytokines playing key roles in learning, memory, and emotional processing. In physiological conditions, cytokines are the main regulators of the brain, thus playing a central role in learning, memory and regulation of the emotions [2,3]. However, the balance of neural and immune activity under physiological conditions can be disrupted by several triggers, thus affecting neuroplasticity, neurotransmitter systems, and neuroendocrine function [4,5]. When sustained inflammatory signalling is maintained, these physio-pathological mechanisms can have detrimental effects, also leading to depression and cognitive impairment [6,7]. Most notably, studies have demonstrated that the dysregulated secretion of cytokines, chemokines, and growth factors are associated with mood disorder [8], also affecting the clinical course [9,10,11], response to pharmacological treatment [12,13,14,15] and brain imaging measures [16,17]. Moreover, cognitive impairment has been associated with inflammation in different clinical populations, such as in patients affected by psychiatric disorders [18,19], neurological disorders [20,21], physical disorders [22,23], and COVID-19 [24,25,26], as well as in the general population [27].

However, despite these individual associations, the interactions between inflammation, depression, and cognition have not been fully explored as an interconnected system, leaving a gap in understanding how these factors jointly contribute to a shared pathophysiology [28,29]. Moreover, in this complex intersection, the interplay between inflammation, depression, and cognition appears to differ between males and females. Indeed, previous findings on sex differences in inflammation-related depressive symptoms have been inconsistent, with some studies suggesting stronger associations in women and others in men [30,31]. Despite these observations, there remains a lack of studies that simultaneously examine the intricate relationships between biomarkers of inflammation, cognitive function, and depressive symptoms, especially when considering the potential role of sex differences.

Notably, COVID-19 patients experience a hyper-inflammatory syndrome, with increased circulating levels of several cytokines, including IL-2, IL-6, IL-10, and TNF-α, and MCP-1 [32]. The literature consistently confirms that COVID-19 patients are largely affected by depressive psychopathology [33] and cognitive impairment [34] to the extent that neuropsychiatric symptoms are listed as a major complication in long COVID syndrome [35]. In this context, investigations in COVID-19 patients have helped increase our understanding of the role of an infective trigger and its associated long-term peripheral inflammation on depression and cognition. In this population, it has been previously observed that both post-COVID depressive psychopathology [25,36,37,38,39] as well as cognitive impairment [25,34] are associated with markers of inflammation, and depressive psychopathology was found to be one of the main risk factors for cognitive impairment [25,40].

Given this background, in the present study, we aimed to investigate the network of inflammatory biomarkers, depressive psychopathology, and cognitive impairment in male and female post-COVID patients. By implementing path modelling analysis techniques, this research will offer a more nuanced understanding of how these factors interact, potentially uncovering differential associations between biomarkers, cognition, and depression in adults of different sexes. For this, we considered a large panel of 48 inflammatory markers, chosen based on their previous associations with diagnosis and cognitive and affective outcomes in mood disorders [10]. Firstly, we investigated the association in the whole sample, to examine the overall system of biomarkers, depression, and cognition. Secondly, considering that we expected that there would be differential associations between biomarkers, cognition, and depression for males and females, we then tested whether the major findings were replicated in male and female subgroups.

## 2. Materials and Methods

### 2.1. Participants and Data Collection

We enrolled 101 COVID-19 survivors during an ongoing prospective study at IRCCS San Raffaele Hospital in Milan, providing biobanking for COVID-19 research.

Diagnosis of COVID-19 was ascertained through radiological findings obtained at the emergency department and further confirmed via reverse transcriptase polymerase chain reaction assays on the nasopharyngeal, throat, or lower respiratory tract swab.

Participants underwent immune–inflammatory profiling as well as depressive symptomatology screening one month after hospital discharge, while neuropsychological assessment was administered at three-month follow-up, in the context of multidisciplinary follow-ups at the outpatient COVID-19 clinic. Only those discharged from the emergency department with a diagnosis of COVID-19 infection were enrolled.

To keep a naturalistic study design, exclusion criteria were limited to age exceeding the range of 18–70 years, intellectual disability, major medical/neurological disorders, and pregnancy. After a complete description of the study, written informed consent was obtained.

### 2.2. Neuropsychiatric Assessment

At one- and three-month follow-up, neuropsychiatric evaluation was performed in an outpatient setting by the psychiatrists in charge using an unstructured psychiatric interview and validated self-report questionnaires.

Depressive symptomatology was rated according to Zung Severity Rating Scale (ZSDS) and to the Beck Depression Inventory (BDI-13). The ZSDS is a 20-item straightforward instrument to measure the presence and severity of depression due to its design based on the diagnostic criteria for depression [41]. The ZSDS showed high sensitivity to identify clinically relevant depression and the need for antidepressant treatment in COVID-19 survivors [30]. The BDI total score reflects the cognitive, affective, somatic, and vegetative symptoms of depression [42]. Besides showing optimal performance in both clinical and non-clinical samples [43], BDI-13 is proven to be an effective tool for rating depressive symptomatology in post COVID-19 patients as well [44]. Altogether, both tools showed high sensitivity for detecting longitudinal changes in post-COVID-19 depression severity, also in relation to the pattern of change in systemic inflammation burden during the illness course [25]. Standard cut-off scores were used to consider the presence of clinically relevant depressive psychopathology (ZSDS index ≥ 50; BDI-13 ≥ 9).

Moreover, at three-month follow-up, the Brief Assessment of Cognition in Schizophrenia (BACS) was implemented to perform a neuropsychological assessment of COVID-19 survivors. This battery consists of six trials including the following: list learning for the evaluation of verbal memory, a verbal fluency task, digit sequencing for working memory, a symbol-coding trial assessing selective attention, token test for psychomotor coordination, and Tower of London testing executive functions. Raw scores can be easily converted into adjusted values considering age, education and gender as confounders, according to normative values correction grids [45]. This tool exhibited high-performance in detecting cognitive dysfunction in COVID-19 survivors [25,40] both at mid- and long-term follow-ups. In the current study, adjusted scores in each subtest were entered as variables of interests in the analysis.

### 2.3. Laboratory Determinants

Bio-Plex Pro Human Cytokine 48-Plex Panel assay (BIO-RAD) was used to detect plasma concentrations of immune analytes, through the bead-based Luminex system, according to xMAP technology (Luminex 200ä system, Merck Millipore, Darmstadt, Germany). This system allows 48 cytokine and chemokine cell signalling molecules to be detected as follows: FGF-basic, Eotaxin, G-CSF, GM-CSF, IFN-γ, IL-1β, IL-1ra, IL-1α, IL-2Rα, IL-3, IL-12 (p40), IL-16, IL-2, IL-4, IL-5, IL-6, IL-7, IL-8, IL-9, GRO-α, HGF, IFN-α2, LIF, MCP-3, IL-10, IL-12 (p70), IL-13, IL-15, IL-17A, IP-10, MCP-1, (MCAF), MIG, β-NGF, SCF, SCGF-β, SDF-1α, MIP-1α, MIP-1β, PDGF-BB, RANTES, TNF-α, VEGF, CTACK, MIF, TRAIL, IL-18, M-CSF, TNF-β. This multiplexed sandwich immunoassay was developed from commercially available capture and detection antibodies and standard proteins, validated and approved by EDI-GMBH. Luminex experiments were performed according to the pre-optimized protocol provided by the manufacturer. The intra-assay coefficient of variation relative to 48-Plex was X%, while the inter-assay coefficient of variation was X%. Analyses were performed on observed concentrations (pg/mL) calculated using Belysa Immunoassay Curve-Fitting Software (version 1.2).

### 2.4. Statistical Analyses

To disentangle the complex pattern of association between inflammatory markers, depressive symptomatology and long-term cognitive dysfunction, we exploited the partial least-squares path modelling (PLS-PM) technique, as provided by the R package plspm [46]. This approach is a powerful multivariate statistical method, achieving optimal statistical power even when high-dimensional data co-exist with limited sample size [47]. The path model consists of two layers as follows: the set of measurements blocks, also known as manifest variables (MVs) or indicators, and the set of relationships among the so-called latent variables (LVs). Essentially, it allows causal pathways between constructs, whose association strength is proxied by path coefficients as inner model metrics, to be explored. In addition, PLS-PM provides estimates of latent–manifest feature causal linkage in a reflective (Mode A) or formative (Mode B) way according to the construct’s nature. Outer model parameters entail factor loadings and weights as valuable metrics for the absolute and relative contribution of an indicator to its construct. Latent factors properties were inspected by computing the variance inflation factor (VIF) of corresponding emergent features, thus ensuring appropriate measurement model definition.

Given our hypothesis, we modelled the inner path diagram by setting the whole 48-plex panel, as an indicator of latent variable inflammation, as the exogenous panel, predicting both Depression as a latent factor and Cognition as a latent variable, the former consisting of ZSDS-index and BDI-13 total scores and the latter indicated by the six-domain adjusted scores of BACS battery. Finally, the putative influence of Depression on Cognition was also considered in the inner model design. We then explored sex-disparities in the PLS-PM model, by specifically comparing matching path coefficients of inner diagrams in the groups of males and females. Function plspm.groups was run for that purpose, returning *t*-test and corresponding *p*-values metrics. When the presence of significant differences in path coefficients was detected, separate PLS-PM models were performed.

For each PLS-PM outer model and for each block, confidence intervals generated from 1000 bootstrap resampling were used to determine the R2 coefficients of determination significance for endogenous variables in the inner models, as well as for indicators’ loading significance in building latent variables. According to the study of [48], the loading factor should be above 0.7 for interpretation purposes. The goodness-of-fit (GoF) metric was used to evaluate overall inner model performance.

Given that PLS-PM does not allow confounders to be considered in the modelling, we additionally employed a two-stage regression approach. First, the inflammation block was separately regressed on age as potential confounder, and second, PLS-PM was fit on the obtained residuals of the inflammation block, together with depressive scores and adjusted values for BACS battery.

## 3. Results

Socio-demographics, clinical features, and inflammatory markers of the sample were resumed in Table 1.

**Figure 1:**
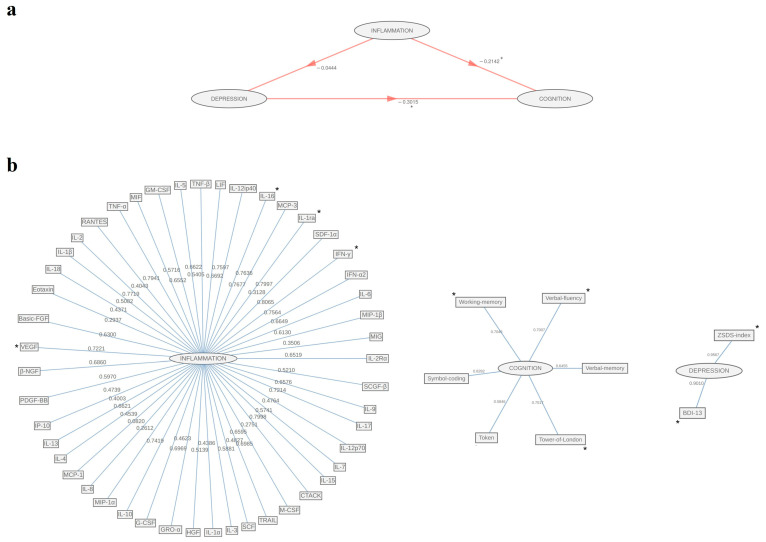
Graphical representation of PLS-PM among the inflammation, depression, and cognition blocks in the whole sample: (**a**) inner model path coefficients among blocks; (**b**) outer model loadings of original features on the corresponding inner block. Significant relationships are marked with *.

Path coefficients of the whole-group PLS-PM inner model are shown in Figure 1a. Inflammation has no effect on Depression in the whole cohort, while inner diagram revealed the significantly negative impact of Inflammation on Cognition (β = −0.21, t = −2.27, *p* = 0.025), together with a marked negative effect of Depression on Cognition (β = −0.3, t = −3.2, *p* = 0.002). Overall, the model achieved moderate performance (GoF = 0.17). Moreover, 1000 bootstrap sampling returned significant coefficients of determination for both Depression (R2 = 0.05, 95% CI: 0.002, 0.159) and Cognition (R2 = 0.2, 95% CI: 0.084, 0.342).

In the outer model, loadings display quantitative relationships between indicators and the corresponding latent constructs (Figure 1b). For Inflammation block, we detected compelling positive contributions of growth factors VEGF (loading = 0.72, 95% CI: 0.008, 0.845); Cytokines IL-16 (loading = 0.76, 95% CI: 0.193, 0.839), IL-1ra (loading = 0.8, 95% CI: 0.036, 0.855), IFN-γ (loading = 0.81, 95% CI: 0.12, 0.881) [49]. Regarding the Depression block, both ZSDS-index (loading = 0.96, 95% CI: 0.912, 0.984) and BDI-13 (loading = 0.96, 95% CI: 0.826, 0.961) reached statistical significance. For its part, results for the latent variable, Cognition, significantly affected four domains of BACS: verbal fluency (loading = 0.7, 95% CI: 0.486, 0.81), working memory (loading = 0.7, 95% CI: 0.521, 0.818), symbol coding (loading = 0.83, 95% CI: 0.691, 0.883), executive functions (loading = 0.7, 95% CI: 0.494, 0.854) (Table 2).

Next, PLS-PM models comparison between sexes revealed significant differences between pairs of path coefficients.

**Figure 2:**
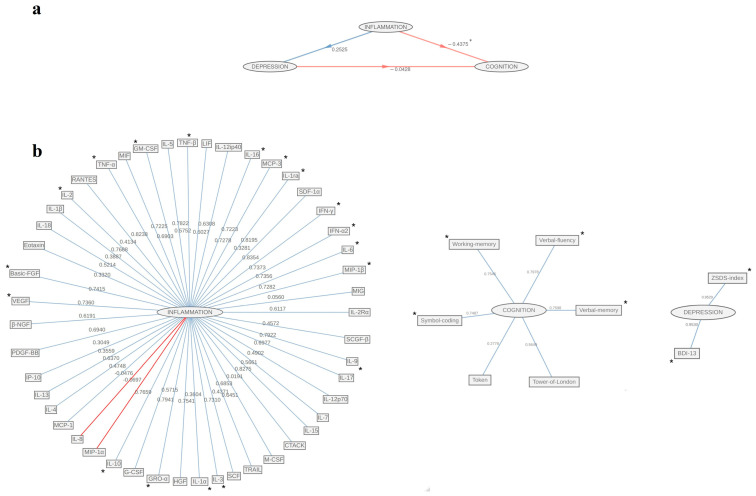
Graphical representation of PLS-PM among the Inflammation, Depression, and Cognition blocks in the female subgroup: (**a**) inner model path coefficients among blocks; (**b**) outer model loadings of original features on the corresponding inner block. Significant relationships are marked with *.

Firstly, the discrepancy between path coefficients of Inflammation and Depression was statistically significant between groups (females vs. males: t = 3.01, *p* = 0.003), as well as those demonstrating Inflammation—Cognition linkage (females vs. males: t = −2.6, *p* = 0.011), whereas the difference in the association between the Depression and Cognition blocks did not reach statistical significance, although it was close (females vs. males: t = 1.89, *p* = 0.062). In females, we uncovered a significant negative effect of Inflammation on Cognition (β = −0.44, t = −3.07, *p* = 0.004), whereas it did not affect Depression (β = 0.25, t = 1.71, *p* = 0.094). Finally, the association of Depression with Cognition was not significant in females (β = −0.04, t = −0.30, *p* = 0.765), (Figure 2a).

The inner model achieved good performance, as proxied by GoF = 0.23, while R2 for the two endogenous variables was equal to 0.06 (95% CI: 0.001, 0.382) and to 0.2 (95% CI: 0.151, 0.532) for Depression and Cognition, respectively. In this group, for the Inflammation block, the 1000 bootstrap iterative sampling led to the following significant cytokines (Figure 2b): IL-16 (loading = 0.72, 95% CI: 0.475, 0.817), IL-3 (loading = 0.82, 95% CI: 0.268, 0.888), IL-6 (loading = 0.74, 95% CI: 0.736, 0.887), IFN-α2 (loading = 0.74, 95% CI: 0.737, 0.872), IL-1α (loading = 0.75, 95% CI: 0.389, 0.876), IL-10 (loading = 0.77, 95% CI: 0.471, 0.902), IL-2 (loading = 0.77, 95% CI: 0.364, 0.364), IL-9 (loading = 0.79, 95% CI:0.364, 0.888), IL-1ra (loading = 0.82, 95% CI: 0.545, 0.902), TNF-α (loading = 0.82, 95% CI: 0.556, 0.898), IL-15 (loading = 0.83, 95% CI: 0.449, 0.915), IFN-γ (loading = 0.84, 95% CI: 0.569, 0.907); growth factors: GM-CSF (loading = 0.72, 95% CI: 0.499, 0.883), MCP-3 (loading = 0.73, 95% CI: 0.728, 0.87), MIP-1β (loading = 0.728, 95% CI: 0.485, 0.837), VEGF (loading = 0.736, 95% CI: 0.321, 0.876), Basic-FGF (loading = 0.74, 95% CI: 0.387, 0.872); Chemokines: GRO-α (loading = 0.79, 95% CI: 0.561, 0.895). Considering Cognition indicators, selective attention (loading = 0.75, 95% CI: 0.05, 0.883), working memory (loading = 0.75, 95% CI: 0.185, 0.881), verbal memory (loading = 0.76, 95% CI: 0.068, 0.871), and verbal fluency (loading = 0.8, 95% CI: 0.145, 0.88) substantially contribute to the construct. Both ZSDS scores (loading = 0.952, 95% CI: 0.701, 0.998) and BDI-13 (loading = 0.95, 95% CI: 0.674, 0.998) were significantly associated with Depression (Table 3).

**Figure 3:**
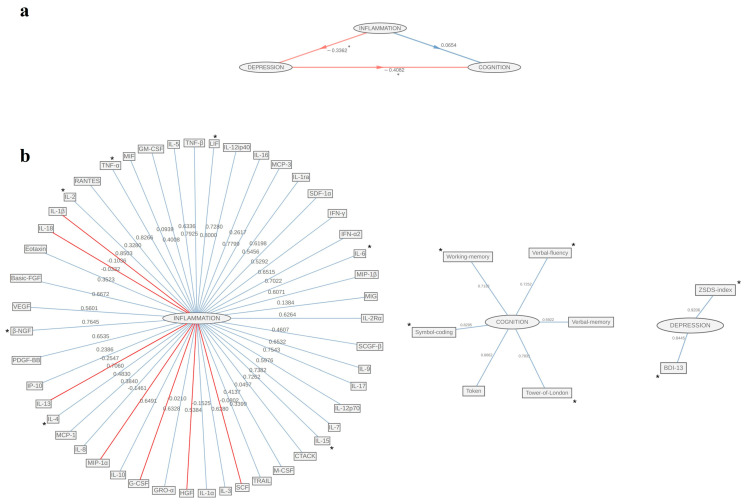
Graphical representation of PLS-PM among the inflammation, depression, and cognition blocks in the male subgroup: (**a**) inner model path coefficients among blocks; (**b**) outer model loadings of original features on the corresponding inner block. Significant relationships were marked with *.

Conversely, in males, a strong negative association was observed between the Inflammation block and Depression (β = −0.336, t = −2.62, *p* = 0.011), as well as for Depression and Cognition linkage (β = −0.41, t = −3.11, *p* = 0.003), while the effect of Inflammation on Cognition was no longer significant (β = 0.065, t = 0.5, *p* = 0.621) (Figure 3a).

GoF was 0.22, with R2 equal to 0.12 (95% CI: 0.003, 0.376) for Depression and R2 equal to 0.19 (95% CI: 0.14, 0.5) for Cognition. The first block showed significant positive loadings for cytokines (Figure 3b): IL-6 (loading = 0.7, 95% CI: 0.004, 0.869), IL-4 (loading = 0.71, 95% CI: 0.001, 0.841), IL-15 (loading = 0.73, 95% CI: 0.117, 0.908), LIF (loading = 0.73, 95% CI: 0.062, 0.873), TNF-α (loading = 0.83, 95% CI: 0.014, 0.92), IL-2 (loading = 0.92, 95% CI: 0.005, 0.903); growth factors β-NGF (loading = 0.76, 95% CI: 0.087, 0.919). Cognition latent factor was driven mainly by selective attention (loading = 0.83, 95% CI: 0.667, 0.892), working memory (loading = 0.71, 95% CI: 0.44, 0.832), verbal fluency (loading = 0.73, 95% CI: 0.725, 0.847), executive functions (loading = 0.78, 95% CI: 0.613, 0.871). As before, both manifest variables of Depression were significantly represented by the construct, BDI-13 (loading = 0.84, 95% CI: 0.554, 0.939), ZSDS (loading = 0.92, 95% CI: 0.855, 0.992) (Figure 3b, Table 3).

## 4. Discussion

The present findings elucidated the relationship between inflammation, depressive psychopathology, and cognitive impairment in post-COVID patients, particularly when exploring the effect of sex.

In the whole group of patients, we found that cognitive functioning was negatively affected both by inflammatory mediators (VEGF, IL-16, IL-1ra, IFN-γ) and by depressive psychopathology. Then, more interestingly, different path coefficients between males and females were highlighted. In females, we observed a significant negative effect of several inflammatory mediators (IL-1α, IL-2, IL-3, IL-6, IL-9, IL-10, IL-15, IL-16, IL-1ra, TNF-α, IFN-α2, IFN-γ, MCP-3, MIP-1β, GRO-α, VEGF, Basic-FGF, and GM-CSF) on cognitive functioning, and a marginal, though not statistically significant, association between the same inflammatory markers and greater depressive psychopathology. In males, the inflammatory mediators (IL-2, IL-4, IL-6, IL-15, LIF, TNF-α, β-NGF) showed a significant negative association with depressive psychopathology severity, and depressive psychopathology was associated with poorer cognitive functioning. The present findings elucidate how sex-specific factors, by affecting immune functioning and subsequent inflammation levels, can influence vulnerability to depressive psychopathology and cognitive impairment [50]. Notably, depression is at least twice as common in women as it is in men [51]. Research suggests that biological factors linked to sex steroid hormones and inflammation [52] play a role in this disparity. Sex steroid hormones can affect inflammation levels by modulating the expression of immune factors, including those involved in initiating immune responses, monitoring the immune system, and maintaining immune activity to counter pathogens [52,53].

Previous studies have found sex differences in the relationship between inflammation and depression. Women were found to be more vulnerable to inflammation-induced mood and behaviour changes [54,55]. Moreover, depression symptom severity, as well as specific symptoms including cognitive symptoms, interest activity, and suicidality correlated with CRP levels only among females [56]. In clinical populations, IL-6, IL-1β, and CRP were elevated among women with depression, whereas these markers were not elevated in men, and rather men displayed elevated levels of IL-17 [57]. Again, it was found that CRP predicted worsening depression in women, but not in men, while depressive symptoms predicted increasing inflammation for men, but not for women [31]. A relevant role of sex hormones was hypothesized to sustain this disparity, and, interestingly, men with depression were found to present with lower testosterone (not exhibiting anti-inflammatory properties) and higher CRP levels compared to male healthy controls [58,59]. Interestingly, in women, it was observed that genetic factors related to inflammation and estradiol predicted post-partum depression, which was also associated with abnormalities in basal ganglia volume [60,61].

Consistent with the reported literature, we observed that specific inflammatory mediators negatively affected cognition and depressive psychopathology only in women, while other mediators showed a protective effect in men. Our results, by exploring a broad spectrum of inflammatory mediators, deepen current knowledge. In fact, contrary to previous studies, we considered not only pro-inflammatory mediators, but a complete panel of inflammatory markers composed of pro-inflammatory cytokines, regulatory cytokines, chemokines, and growth factors. In men, the immune/inflammatory mediators (IL-2, IL-4, IL-6, IL-15, LIF, TNF-α, β-NGF+) found to protect against depressive psychopathology were mainly related to immune regulation, growth, and maintenance. IL-2 specifically expands and activates CD4+ Treg cell populations and can control inflammation [62,63,64], acts directly as a trophic factor on both neurons and oligodendrocytes [65], and showed a significant antidepressant effect by expanding the population of Treg, Th2, and Naive CD4+/CD8 + immune cells [66]. IL-4 plays an important role in regulating antibody production, haematopoiesis, and inflammation [67]. In the brain, the production of IL-4 plays a primary role in restoring balanced CNS function and cognition after injury [68]. IL-15 presents immunomodulatory effects on cells of both the innate and adaptive immune systems, which play a central role in defence mechanisms against pathogens [69]. In the brain, IL-15 modulates neurotransmission, facilitating mood stability that helps to limit metabolic consequences during a neuroinflammatory challenge with LPS [70]. β-NGF is a neurotrophin primarily involved in the growth, maintenance, proliferation, and survival of peripheral and central neurons [71], with a modulatory factor in the hypothalamic–pituitary–adrenal axis [72]. A recent meta-analysis also confirmed that MDD patients showed significantly lower peripheral NGF levels than those in HCs [73]. LIF plays a crucial role in the pro-survival and anti-inflammatory effects of IL-6 cytokines, increasing its activity and expression and promoting tissue repair and better outcomes in models of neurodegeneration and inflammation [74]. LIF exerts pleiotropic effects on several cell populations of the CNS by exerting wide-ranging effects on cellular survival, maintenance, and development [74]. IL-6 and TNF-α are well known for their pro-inflammatory effects; however, these mediators also exhibit context-dependent immune-regulatory activities, being required for many aspects of vital CNS function, such as synaptic scaling, ensuring functional LTP in the hippocampus, triggering neuron survival after injury [2,68,75,76].

In women, on the contrary, cognitive impairment was significantly predicted by a mixed anti- and pro-inflammatory profile, while a similar, though not statistically significant, association was observed for depressive psychopathology—suggesting a dysregulated immune–inflammatory response. In detail, the involved inflammatory markers were pro-inflammatory mediators (IL-1α, IL-6, IFN-α2, IFN-γ, TNF-α,), anti-inflammatory mediators (IL-10, IL-1ra), pleiotropic cytokines (IL-2, IL-9, L-15, IL-16, IL-3), growth factors (VEGF, Basic-FGF, and GM-CSF), and chemoattractant inflammatory chemokines (MCP-3, MIP-1β, GRO-α) [77,78]. Thus, in women, we observed a more dysregulated immune/inflammatory response involving several mediators with mixed functions.

Taking together the findings observed in men and women, it seems that different sex-specific immune/inflammatory mechanisms underlie the interactive networks of depression and cognition in post-COVID patients. In women, SARS-CoV-2 infections induce systemic inflammation sustained by several heterogeneous inflammatory mediators with a main pro-inflammatory profile that persists months after infection and is associated with depression and cognitive impairment. In men, on the other hand, it seems that after infection and acute disease, the immune/inflammatory set-point turns on a regulative profile able to resolve the acute inflammatory status and to protect from subsequent depression. We speculate that sex-specific endocrine mechanisms sustain this different inflammatory response and may be responsible for the epidemiological imbalance of depression between men and women [79,80].

Strengths of the present study are its naturalistic design in a specialized clinical setting, and state-of-the-art analytical methods, but we acknowledge some limitations. The limited health care resources and patient’s compliance related to the clinical setting forced us to choose self-rating scales instead of a structured clinical interview to assess depression. Recruitment was in a single centre, thus raising the possibility of population stratification. Additionally, the lack of a healthy control group prevents us from drawing definite conclusions about the pattern of immune alterations underlying neuropsychiatric issues in post-acute COVID-19 stages.

Despite these limitations, this study adds several elements to the current understanding of the immune–molecular mechanisms underlying viral-induced neuropsychiatric manifestations, providing a possible physio-pathological explanation of the well-known sex disparities in depressive psychopathology.

## 5. Conclusions

These findings highlight the growing importance of recognizing sex differences in immunological and neuropsychiatric responses to COVID-19. Future studies should further explore how hormonal and immune interactions may inform personalized, sex-specific treatment approaches in post-COVID care.

## Figures and Tables

**Table 1:** Socio-demographic, clinical, and inflammatory profiles of participants according to sex. Reported values are Mean ± SD, and Median ± IQR, respectively. Levels of significance were determined according to Student’s *t* test and Chi-square for sociodemographic and neuropsychiatric information and Mann–Whitney test for inflammatory data. *, **, and *** for *p* < 0.05, <0.01, <0.001.

Socio-Demographics and Clinical Features	Whole Sample (*n* = 101)	Females (*n* = 45)	Males (*n* = 56)	t or χ^2^	*p*-Values
Males (females)	56 (45)	-	-	-	-
Age	53.23 ± 10.3	51.78 ± 11.46	54.39 ± 9.2	−1.27	0.206
Education (years)	12.98 ± 3.60	12.67 ± 3.88	12.73 ± 3.59	0.09	0.93
BDI-13	4 ± 5.57	5.76 ± 6.39	2.68 ± 4.41	2.86	0.005 **
BDI-13 ≥ 9 yes (%)	13 (12.87%)	9 (20%)	4 (7.14%)	3.68	0.055
ZSDS index	46.18 ± 12.61	52.34 ± 12.64	41.23 ± 10.26	4.88	<0.001 ***
ZSDS index ≥ 50 (%)	37 (36.63%)	24 (53.34%)	13 (23.21%)	9.75	0.002 **
BACS—verbal memory	49.64 ± 9.75	51.59 ± 10.33	48.07 ± 9.05	1.82	0.071
BACS—verbal fluency	46.8 ± 12.16	43.99 ± 9.42	47.76 ± 13.84	−1.56	0.121
BACS—working memory	21.16 ± 5.17	20.08 ± 4.89	22.03 ± 5.27	−1.92	0.058
BACS—selective attention	50.97 ± 11.97	50.88 ± 12.06	51.04 ± 12	−0.06	0.949
BACS—psychomotor coordination	72.63 ± 19.34	71.99 ± 17.23	73.14 ± 21.03	−0.3	0.768
BACS—executive functions	14.75 ± 4.55	14.4 ± 4.59	15.03 ± 4.55	−0.7	0.489
**Immune-Inflammatory Biomarkers**	**Whole Sample** **(*n* = 101)**	**Females** **(*n* = 45)**	**Males (*n* = 56)**	**Z**	***p***
IL-2Rα	32 ± 11	30 ± 9	35 ± 11.75	−2.34702	0.019 *
MIG	45 ± 39	41 ± 45	52 ± 32.5	−1.32895	0.183864
MIP-1β	430 ± 90	405 ± 85	437.25 ± 86	−1.56468	0.117659
IL-6	15 ± 4	14.5 ± 4	15.75 ± 4.25	−1.34262	0.179396
IFN-α2	15 ± 2	15 ± 3	16 ± 2.75	−1.41778	0.156256
IFN-gamma	38 ± 9	36 ± 9	39.25 ± 7.625	−1.81066	0.070195
SDF-1α	283.5 ± 143.5	255 ± 137	307.5 ± 147.5	−1.94048	0.052323
IL-1ra	10 ± 3.5	10 ± 4	10 ± 3	−0.50562	0.613126
MCP-3	18 ± 3	18 ± 2	18 ± 3	−0.84383	0.398763
IL-16	22 ± 6	21 ± 5	24 ± 7	−2.18645	0.028783 *
IL-12(p40)	14 ± 2	14 ± 1.75	14 ± 3	−1.52368	0.127588
LIF	17 ± 3	16 ± 3	17 ± 2.875	−1.77308	0.076217
TNF-β	163 ± 38.5	158 ± 29	164.75 ± 36.5	−1.45877	0.144628
IL-5	26.5 ± 8	24 ± 7	27.5 ± 8	−2.10446	0.035339 *
GM-CSF	16 ± 3	16 ± 3	16.75 ± 3.75	−0.37921	0.704530
MIF	409 ± 153	394 ± 147.5	451 ± 149.75	−0.96341	0.335345
TNF-α	21 ± 5.5	21 ± 6	21 ± 4.25	−0.77551	0.438040
RANTES	2463 ± 772	2389 ± 657.5	2564.25 ± 853.625	−1.07615	0.281863
IL-2	12 ± 3	12 ± 2	12 ± 3	−0.74134	0.458485
IL-1β	13 ± 5	12 ± 6	13 ± 5	−0.98390	0.325163
IL-18	52 ± 24.5	47.75 ± 21	57.5 ± 25.25	−2.32994	0.019810 *
Eotaxin	223.5 ± 106.5	199 ± 106.5	238.75 ± 124	−2.48367	0.013004 *
Basic_FGF	13 ± 2.5	12.5 ± 3	13 ± 3	−1.99514	0.046029 *
VEGF	28 ± 6.25	28 ± 6	29 ± 6.75	−1.08981	0.275797
β-NGF	15.5 ± 3	15.5 ± 3	15.75 ± 3.5	−0.11957	0.904823
PDGF-BB	35 ± 15.5	31.5 ± 14	38 ± 13.25	−1.91315	0.055730
IP-10	142 ± 97	123 ± 99.5	154.25 ± 115	−2.35044	0.018752 *
IL-13	18 ± 8	18 ± 11	18 ± 5.5	0.02733	0.978196
IL-4	14 ± 3.5	14 ± 3.5	14.25 ± 3.25	−1.40411	0.160286
MCP-1	32 ± 16	30 ± 11	34 ± 16.25	−2.09421	0.036242 *
IL-8	15 ± 4	14.5 ± 5.25	15 ± 3.75	−0.49878	0.617931
MIP-1α	16 ± 5	16.5 ± 4	16 ± 6	−0.60811	0.543117
IL-10	10 ± 3	10 ± 2	10 ± 3	−1.07273	0.283394
G-CSF	20 ± 3.5	19.5 ± 3	20 ± 4.25	−0.71743	0.473109
GRO-α	46 ± 13.5	46.5 ± 15	45.5 ± 12.5	−0.04441	0.964576
HGF	37 ± 12.5	37 ± 12	37 ± 13	−0.45779	0.647105
IL-1α	12.5 ± 2	12 ± 2	13 ± 2	−1.42803	0.153285
IL-3	17 ± 4	17 ± 3	17 ± 3	−0.93608	0.349235
SCF	29 ± 9.5	29 ± 11	29.5 ± 9.5	−0.89508	0.370745
TRAIL	29 ± 6.5	29 ± 8.5	28 ± 6.5	0.43046	0.666863
M-CSF	34 ± 7.5	32 ± 6	35.75 ± 7.25	−2.26161	0.023722 *
CTACK	99 ± 43	98 ± 49	100 ± 43.75	−0.91558	0.359889
IL-15	20 ± 5	20 ± 4	21 ± 4.5	−1.19572	0.231808
IL-7	14 ± 3	13 ± 2	14 ± 3.75	−2.43243	0.014998 *
IL-12(p70)	19 ± 4	18 ± 3	19 ± 5.25	−1.82091	0.068622
IL-17	15 ± 2	14 ± 3	15 ± 2	−1.63984	0.101039
IL-9	120 ± 25.25	113 ± 24	121.75 ± 25.75	−1.04881	0.294264
SCGF-β	68 ± 28.5	62.5 ± 29	72 ± 28.5	−1.55102	0.120899

**Table 2:** Outer model loadings for Inflammation, Cognition, and Depression blocks in the whole sample. Original loadings and 95% confidence intervals (CI) were reported for each variable. Significant variables exceeding the 0.7 threshold are marked in bold.

Whole Sample (*n* = 101)			
Inflammation			
Variables	Original Loadings	Boot Lower CI	Boot Upper CI
IL-8	0.082	−0.137	0.673
MIP-1α	0.261	−0.099	0.437
CTACK	0.275	−0.14	0.566
Eotaxin	0.294	−0.236	0.511
SDF-1α	0.313	−0.292	0.605
MIG	0.351	−0.057	0.55
IL-13	0.401	−0.215	0.75
RANTES	0.404	0.0731	0.632
IL-18	0.437	0.032	0.653
HGF	0.439	−0.172	0.731
MCP-1	0.454	−0.198	0.606
G-CSF	0.462	0.051	0.809
IP-10	0.474	−0.005	0.641
IL-12p70	0.476	−0.383	0.72
SCF	0.483	−0.044	0.69
IL-1β	0.508	−0.081	0.721
IL-1α	0.514	−0.221	0.851
SCGF-β	0.521	−0.073	0.685
IL-5	0.54	−0.346	0.779
GM-CSF	0.572	0.069	0.781
IL-7	0.575	−0.345	0.785
IL-3	0.588	−0.29	0.795
PDGF-BB	0.597	0.02	0.776
MIP-1β	0.613	0.035	0.778
Basic-FGF	0.63	−0.247	0.842
IL-2Rα	0.652	−0.015	0.838
MIF	0.655	0.145	0.842
IL-9	0.658	0.021	0.814
M-CSF	0.659	0.042	0.819
IL-4	0.662	−0.246	0.796
TNF-β	0.662	0.074	0.815
IL-6	0.665	−0.248	0.834
IL-12ip40	0.669	−0.334	0.83
β-NGF	0.686	−0.038	0.85
GRO-α	0.697	0.121	0.856
TRAIL	0.699	0.129	0.813
IL-17	0.721	−0.212	0.844
VEGF	**0.722**	**0.008**	**0.845**
IL-10	0.742	−0.109	0.826
IFN-α2	0.756	−0.13	0.841
LIF	0.76	−0.18	0.837
IL-16	**0.763**	**0.192**	**0.839**
MCP-3	0.768	−0.173	0.858
IL-2	0.772	−0.244	0.889
TNF-α	0.794	−0.05	0.883
IL-1ra	**0.8**	**0.036**	**0.855**
IL-15	0.8	−0.058	0.883
IFN-γ	**0.806**	**0.12**	**0.881**
**Cognition**			
BACS—Psychomotor coordination	0.585	0.211	0.756
BACS—Verbal memory	0.646	0.219	0.779
BACS—Verbal fluency	**0.701**	**0.485**	**0.81**
BACS—Executive functions	**0.702**	**0.494**	**0.854**
BACS—Working memory	**0.705**	**0.521**	**0.818**
BACS—Selective attention	**0.828**	**0.691**	**0.883**
**Depression**			
BDI-13	**0.901**	**0.826**	**0.961**
ZSDS	**0.957**	**0.912**	**0.984**

**Table 3:** Outer model loadings for Inflammation, Cognition, and Depression blocks according to the biological sex. Original loadings and 95% confidence intervals (CI) were reported for each variable. Variables exceeding 0.7 threshold are marked in bold.

Females (*n* = 45)				Males (*n* = 56)		
Inflammation						
Variables	Original Loadings	Boot Lower CI	Boot Upper CI	Original Loadings	Boot Lower CI	Boot Upper CI
Basic-FGF	**0.742**	**0.387**	**0.872**	0.667	−0.093	0.891
CTACK	0.019	−0.387	0.339	0.046	−0.34	0.721
Eotaxin	0.332	−0.121	0.701	0.352	−0.151	0.572
G-CSF	0.571	0.235	0.787	−0.021	−0.308	0.803
GM-CSF	**0.723**	**0.499**	**0.883**	0.094	−0.357	0.705
GRO-α	**0.794**	**0.561**	**0.895**	0.633	0.083	0.854
HGF	0.360	−0.065	0.673	−0.153	−0.585	0.787
IFN-α2	**0.737**	**0.424**	**0.872**	0.651	0.082	0.838
IFN-γ	**0.835**	**0.569**	**0.907**	0.529	0.053	0.885
IL-10	**0.766**	**0.471**	**0.902**	0.649	0.016	0.832
IL-12ip40	0.603	0.258	0.801	0.8	−0.136	0.839
IL-12p70	0.490	−0.039	0.745	0.598	−0.164	0.772
IL-13	0.356	−0.020	0.672	−0.255	−0.683	0.732
IL-15	**0.827**	**0.449**	**0.915**	**0.726**	**0.116**	**0.908**
IL-16	**0.722**	**0.475**	**0.817**	0.262	−0.276	0.851
IL-17	0.698	0.303	0.868	0.754	−0.007	0.855
IL-18	0.521	0.096	0.819	−0.033	−0.475	0.636
IL-1ra	**0.820**	**0.545**	**0.902**	0.62	0.085	0.854
IL-1α	**0.754**	**0.389**	**0.876**	0.538	−0.064	0.895
IL-1β	0.389	0.075	0.720	−0.104	−0.594	0.737
IL-2	**0.769**	**0.364**	**0.887**	**0.85**	**0.005**	**0.903**
IL-2Rα	0.612	0.088	0.865	0.626	0.036	0.894
IL-3	**0.731**	**0.268**	**0.888**	0.628	−0.089	0.793
IL-4	0.637	0.331	0.791	**0.706**	**0.001**	**0.841**
IL-5	0.575	0.020	0.823	0.793	−0.2	0.874
IL-6	**0.736**	**0.234**	**0.887**	**0.702**	**0.004**	**0.869**
IL-7	0.566	0.190	0.763	0.738	−0.091	0.821
IL-8	−0.048	−0.281	0.744	0.384	−0.04	0.707
IL-9	**0.792**	**0.530**	**0.886**	0.653	0.032	0.827
IP-10	0.305	0.003	0.677	0.239	−0.232	0.682
LIF	0.631	0.308	0.818	**0.728**	**0.062**	**0.873**
MCP-1	0.475	0.098	0.684	0.483	−0.044	0.635
MCP-3	**0.728**	**0.466**	**0.870**	0.78	−0.032	0.877
M-CSF	0.685	0.197	0.883	0.414	−0.089	0.814
MIF	0.690	0.381	0.828	0.401	−0.129	0.855
MIG	0.056	−0.200	0.412	0.138	−0.292	0.724
MIP-1α	−0.070	−0.354	0.290	−0.146	−0.347	0.549
MIP-1β	**0.728**	**0.485**	**0.837**	0.607	0.027	0.78
PDGF-BB	0.694	0.432	0.832	0.653	0.034	0.798
RANTES	0.413	0.087	0.669	0.328	−0.091	0.653
SCF	0.437	0.068	0.683	−0.08	−0.559	0.684
SCGF-β	0.457	0.051	0.710	0.461	0.019	0.742
SDF-1α	0.328	−0.193	0.641	0.546	−0.16	0.735
TNF-α	**0.824**	**0.556**	**0.898**	**0.827**	**0.014**	**0.918**
TNF-β	0.782	0.552	0.882	0.634	0.029	0.808
TRAIL	0.645	0.370	0.780	0.34	−0.183	0.814
VEGF	**0.736**	**0.321**	**0.876**	0.56	0.064	0.871
β-NGF	0.619	0.305	0.839	**0.765**	**0.087**	**0.919**
**Cognition**						
BACS—Executive functions	0.565	−0.311	0.839	**0.783**	**0.613**	**0.871**
BACS—Psychomotor coordination	0.278	−0.333	0.716	0.666	0.282	0.844
BACS—Selective attention	**0.749**	**0.050**	**0.883**	**0.829**	**0.667**	**0.892**
BACS—Verbal fluency	**0.798**	**0.145**	**0.880**	**0.725**	**0.399**	**0.847**
BACS—Verbal memory	**0.760**	**0.068**	**0.871**	0.592	0.182	0.826
BACS—Working memory	**0.755**	**0.185**	**0.881**	**0.713**	**0.44**	**0.832**
**Depression**						
BDI-13	**0.953**	**0.674**	**0.998**	**0.844**	**0.554**	**0.939**
ZSDS	**0.953**	**0.701**	**0.998**	**0.921**	**0.855**	**0.992**

## Data Availability

The data presented in this study are available on request from the corresponding author due to ethical reasons.
